# The Impact of an Algorithm-Guided Management of Preoperative Anemia in Perioperative Hemoglobin Level and Transfusion of Major Orthopedic Surgery Patients

**DOI:** 10.1155/2013/641876

**Published:** 2013-03-27

**Authors:** Dietmar Enko, Franz Wallner, Achim von-Goedecke, Christa Hirschmugl, Vinzenz Auersperg, Gabriele Halwachs-Baumann

**Affiliations:** ^1^Department of Laboratory Medicine, Central Hospital Steyr, Sierningerstraße 170, 4400 Steyr, Austria; ^2^Department of Anesthesiology and Intensive Care, Central Hospital Steyr, Sierningerstraße 170, 4400 Steyr, Austria; ^3^Department of Orthopedic Surgery, Central Hospital Steyr, Sierningerstraße 170, 4400 Steyr, Austria

## Abstract

The aim of this study was to evaluate a laboratory-guided therapeutic algorithm of preoperative anemia. 335 patients with elective hip or knee arthroplasty were included in this retrospective before-after study. Group I (*n* = 101) underwent conventional preoperative procedures before algorithm implementation. Group II (*n* = 234) underwent algorithm-guided preoperative anemia management. A hemoglobin-level of 13 g/dL was the therapeutic cut-off for men and women. Reticulocyte hemoglobin content (CHr) and soluble transferrin receptor (sTfR)/log ferritin ratio were used in the form of the Thomas plot. Iron deficiency (ID) was substituted with 1000 mg iron intravenous (i.v.) and 10000 international units (I.U.) of erythropoiesis-stimulating agent (ESA) subcutaneous (s.c.) or i.v., anemia of chronic disease (ACD) (without functional ID) with 40000 I.U. ESA s.c. or i.v and additionally 200 mg iron i.v. Substituted anemic patients in Group II (*n* = 32) showed a distinctly higher preoperative (Hb-median 13 versus 11.95 g/dL) (*P* < 0.01) and postoperative (Hb-median 9.75 versus 9.0 g/dL) (*P* < 0.05) Hb level compared with untreated anemic patients in Group I (*n* = 24). In Group II red blood cell (RBC) units (35 units/234 patients) were reduced by 44% compared with Group I (27 units/101 patients). Algorithm-guided preoperative anemia management raises perioperative Hb-level and reduces blood use.

## 1. Introduction

Based on the knowledge of the side effects of allogenic blood transfusions, patient blood management (PBM) evolved into a multidisciplinary clinical discipline. 

In the case of complications in hospitalized patients, such as lung injuries or nosocomial infections [1, 2], allogenic blood transfusions are known to be a risk factor. Furthermore, transfusion-related immunomodulation is described [3, 4]. Based on these findings, it is generally agreed that allogenic blood transfusion should be avoided, if possible. Thus, patients should undergo accurate clinical investigation before an elective surgical procedure.

Preoperative anemia management including laboratory diagnosis and therapy of the various forms of anemia is one essential part of PBM. The prevalence of preoperative anemia is positively correlated with the age of the patient and more often found in elderly patients [2, 5]. Anemia of chronic disease (ACD) and iron deficiency (ID) are the most frequent causes of preoperative anemia [2, 6]. Low preoperative hemoglobin (Hb) levels are well known as major predicting factors in requiring perioperative blood transfusion in orthopedic surgery [7, 8]. Patients with preoperative Hb levels between 10 and 13 g/dL may be more often transfused than those with Hb level > 14 g/dL [5, 9].

As far as preoperative laboratory testing is concerned, it is recommended to determine Hb levels in patients with elective orthopedic surgeries 28 days before an intervention [2, 10]. Since anemia is a syndrome with many underlying causes, a diagnostic strategy is required to differentiate the various forms of anemia in the preoperative setting. It is the more important since treatment differs depending on the principle cause of anemia. So, in patients with ACD, treatment with an erythropoiesis-stimulating agent (ESA) is recommended [2, 6, 10, 11]. To potentate the erythropoietic response in erythropoietin therapy and to avoid functional ID, patients should receive intravenous (i.v.) iron supplementation additionally [1, 2, 4, 6, 11, 12]. In contrast, anemia due to ID should be supplemented with i.v. iron in a higher dosage [2, 11]. Additionally preoperative treatment with erythropoietin reduces allogenic blood transfusions [4–6, 9, 11].

The implementation of PBM in the various countries in Europe is inconsistent with a lack of widely accepted guidelines [11]. In order to implement standard operation procedures for preoperative anemia management in our hospital, we established an interdisciplinary group consisting of specialists for laboratory and transfusion medicine, anesthesiology, and orthopedics. The preoperative treatment of anemia was based on the Thomas-plot [13–16]. A threshold of 13 g/dL Hb was used as a cut-off for diagnostic and therapeutic intervention for men as well as women based on published data [5, 17]. This proposed diagnostic strategy was compared with the published Network for Advancement of Transfusion Alternatives (NATA) guidelines [10, 18].

The aim of this study was to investigate the effect of an algorithm-guided preoperative anemia management in perioperative Hb levels and transfusion outcome in orthopedic surgery. 

## 2. Materials and Methods

The ethical approval for this study was provided by the Ethical Committee of Upper Austria, Linz, Austria (study number: K-19-12).

### 2.1. Study Design and Study Groups

**Figure 1:**
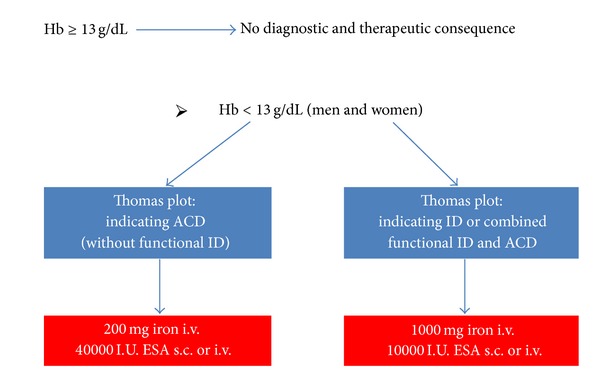
Preoperative diagnostic and therapeutic algorithm applied in Group II. Hb: hemoglobin; ID: iron deficiency; ACD: anemia of chronic disease; ESA: erythropoiesis stimulating agent; I.U.: international unit; s.c.: subcutaneous; i.v.: intravenous.

This study is a retrospective before-and-after study. The study period was from August 1, 2011 to April 15, 2012. As of October 15, 2011 an algorithm of preoperative anemia management was implemented in the context of a PBM program (Figure 1).

**Figure 2:**
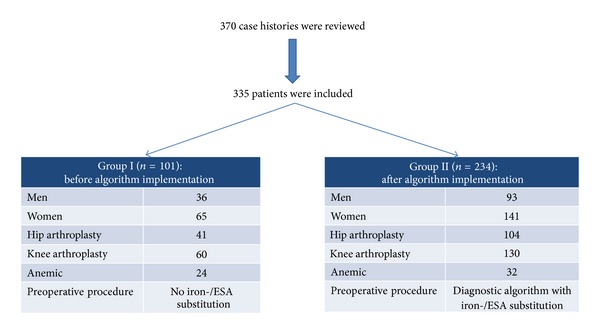
Retrospective before-after study: Group I underwent conventional preoperative procedures without iron-/ESA-substitution before algorithm-implementation. Group II underwent preoperative diagnostic algorithm (Thomas-plot) with preoperative iron-/ESA-substitution. ESA: erythropoiesis stimulating agent.

A total of 370 case histories of patients with elective orthopedic surgery were reviewed. 335 patients were included in this study (Figure 2). 

Inclusion and exclusion criteria: the inclusion criteria were hip or knee arthroplasty in the above-mentioned period and the availability of laboratory parameters at special time points: the first preoperative Hb level up to 3 months before surgery, the second preoperative Hb level immediately before (0–3 days) surgery and at a minimum one Hb-measurement during the first postoperative week. The reasons for the exclusion of 35 patients were the unavailability of pre- and/or postoperative Hb-values, therapeutic intervention within 7 days before surgery, variance from the proposed dosage schedule of iron/ESA substitution, and the postponement or cancellation of scheduled surgery.

Group I (*n* = 101) underwent surgery before the algorithm-implementation in the time between August 1, 2011 and October 15, 2011. If patients showed preoperative anemia (Hb <13 g/dL) in this group, no iron-/ESA-substitution followed.

Group II (*n* = 234) were patients who underwent surgery after an algorithm-implementation in the time between October 15, 2011 and April 15, 2012. Preoperative therapeutic decisions depended on Hb level and results of the Thomas-plot. Hb ≥ 13 g/dL before surgery had no diagnostic and therapeutic consequence. If Hb level was <13 g/dL and results of the Thomas-plot indicated ACD without functional ID, 40000 international units (I.U.) recombinant human erythropoietin (rHuEPO) subcutaneous (s.c.) or intravenous (i.v.) and additionally 200 mg iron i.v. were substituted. If Hb level was <13 g/dL and the Thomas-plot indicated ID or a combination of functional ID and ACD, 1000 mg iron i.v. + 10000 I.U. rHuEPO s.c. or i.v. were substituted.

### 2.2. Thomas Plot, Ferritin and Transferrin Saturation (TSAT)

The Thomas-plot was calculated according to the literature [13–16]. Briefly, the soluble transferrin receptor (sTfR) (reference range: 0.76–1.76 mg/L) and C-reactive protein (CRP) (reference range: 0–3 mg/L) were determined by nephelometric methods, ferritin (reference range: men: 26–388 ng/mL, women: 8–252 ng/mL) by sandwich chemiluminescent immunoassay on Dimension Vista System (Siemens, Vienna, Austria). sTfR and ferritin were used to calculate the sTfR/log ferritin ratio. The cut-off value for this ratio depended on an acute-phase reaction. If CRP was measured ≤5 mg/L, the cut-off was 1.5; if CRP was measured >5 mg/L, the cut-off was 0.8.

**Figure 3:**
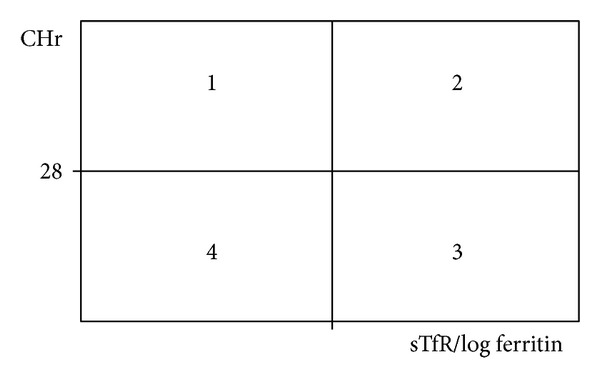
Thomas-plot. Functional ID was defined as a CHr < 28 pg. The cut-off for sTfR/log ferritin ratio depended on the CRP value and was 1.5 for simple ID (CRP ≤ 5 mg/L) and 0.8 for ID combined with acute phase reaction (CRP > 5 mg/L). Quadrant 1 indicated ACD without functional ID, quadrant 2 latent ID, quadrant 3 manifest ID, and quadrant 4 a combined state of functional ID and ACD. CHr: reticulocyte hemoglobin content, sTfR: soluble transferrin receptor, CRP: C-reactive protein, ACD: anemia of chronic disease, ID: iron deficiency.

The reticulocyte hemoglobin content (CHr) (reference range: 28–35 pg) was determined by the measurement of laser light scatter after the isovolumetric sphering of oxazine 750-stained reticulocytes (Advia System, Siemens, Vienna, Austria). CHr < 28 pg indicated functional ID. The relationship between sTfR/log ferritin and CHr is illustrated in Figure 3. Quadrant 1 indicated ACD without functional ID, quadrant 2 latent ID, quadrant 3 manifest ID, and quadrant 4 a combined state of functional ID and ACD. 

In anemic patients in Group II (*n* = 32), the Thomas-plot was compared with diagnostic algorithm including transferrin saturation (TSAT) and ferritin (recommended by NATA guidelines) as discriminating parameters for their use in clinical practice to differentiate between ID and ACD. Transferrin (reference range: 200–360 mg/dL) was determined by the nephelometric method (Dimension Vista System, Siemens, Vienna, Austria). Iron (reference range: men: 65–175 *μ*g/dL, women: 50–170 *μ*g/dL) was determined by the photometric method (Dimension Vista System, Siemens, Vienna, Austria). The percentage of TSAT was calculated based on the formula TSAT (%) = serum iron (*μ*g/dL) × 70.9/transferrin (mg/dL).

### 2.3. Hb-Measurement, MCV, and MCH

The first preoperative clinical investigation and Hb level determination took place in the preoperative outpatient clinic up to 3 months before surgery. The second preoperative Hb level was measured immediately before (0–3 days) surgery, as well as twice more during the first postoperative week. The mean corpuscular volume (MCV) (reference range: 80–99 fl) and the mean corpuscular hemoglobin (MCH) (reference range: 26.4–34 pg) were always measured parallel to Hb levels on Advia System (Siemens, Vienna, Austria). Pre- and postoperative Hb levels in Group I were compared with Group II.

In Group II, in 233 patients first preoperative Hb level was measured with two methods: point-of-care testing (HemoCue, NewMedX, Vienna, Austria) in the preoperative outpatient clinic and the reference method (Advia, Siemens, Vienna, Austria) in the Department of Laboratory medicine. The reliability of the HemoCue hemoglobinometer was assessed compared with the gold standard cyanmethemoglobin measurement on Advia.

### 2.4. Vitamin-B12 and Folic-Acid Measurement

Preoperative vitamin B12 (reference range: 254–1320 pg/mL) and folic acid (reference range: 3.1–17.5 ng/mL) were analyzed with a competitive immunochemiluminescent method (Dimension Vista System, Siemens, Vienna, Austria).

### 2.5. Iron/ESA Preparations and Time Point of Therapeutic Intervention

In Group II iron and rHuEPO were supplemented on an average 20 days before elective surgery. The new i.v. iron preparation ferric carboxymaltose (Ferinject, Vifor Pharma Austria GmbH, Vienna, Austria) was used for iron-substitution. Erythropoietin zeta (Retacrit, Astro-Pharma GmbH, Vienna, Austria) was used for ESA-substitution.

### 2.6. Blood Transfusions

No strict transfusion algorithm for red blood cell (RBC) transfusion was used. If blood transfusions were needed, these were donated allogenic RBC units. The perioperative use of RBC units in Group I was compared with Group II.

### 2.7. Costs

The account costing reflects the therapeutic costs at the time the study was performed. Costs were calculated in Euro (€). The cost account includes the purchase prices of RBC units, i.v. iron preparations, and ESA preparations. The cost elements associated with laboratory testing and staff are not included.

### 2.8. Statistical Analysis

Two-sided 95% confidence intervals (95% CI) were calculated for preoperative courses of iron-related variables. For the time from iron-/ESA-substitution to surgery and the preoperative hemoglobin course Spearman's rank correlation coefficient was calculated. Crohnbach's alpha and Pearson's correlation coefficient were used to test the reliability of hemoglobin measurement by the HemoCue (NewMedX, Vienna, Austria) hemoglobinometer in comparison with the gold standard method on Advia (Siemens, Vienna, Austria). Furthermore the Bland-Altman plot was calculated. For cohort comparisons of metric variables the exact Mann-Whitney *U* test was used. The data of dichotomous variables were compared using the Fisher's exact test, the data of variables with more than two categories were compared by the exact chi-square test. All the tests are two-tailed with a confidence level of 95% (*P* < 0.05). No adjustment for the type I error was made. Therefore the concerning *P* values are only descriptive. 

## 3. Results

### 3.1. Epidemiologic Data and Anemia Prevalence

Of all the reviewed case histories (*n* = 370) with elective orthopedic surgery, 61.9% were women and 38.1% were men. The mean age of patients was 67.73 years ± 9.81. 

Predominantly women showed preoperative anemia. Using the definition of anemia according to the World Health Organization (Hb level < 13 g/dL for men, Hb level < 12 g/dL for women), the anemia prevalence was 7.3% (*n* = 27: 17 women, 10 men). Using a threshold of 13 g/dL as a cut-off for men and women, the prevalence of preoperative anemia was 20% (*n* = 75: 65 women, 10 men). From this, 48 women showed a preoperative Hb level ≥ 12 < 13 g/dL.

### 3.2. Pre- and Postoperative Hb levels (Figure 4)

*Anemic Patients.* Substituted anemic patients in Group II (*n* = 32) presented a distinctly higher preoperative Hb level (median: Hb 13 g/dL (min/max: 10.6/14.7)) immediately before (0–3 days) surgery compared with untreated anemic patients in Group I (*n* = 24) (median: Hb 11.95 g/dL (min/max: 8.8/13.8)) (*P* < 0.01). 

Furthermore substituted anemic patients in Group II retained a higher postoperative Hb level. The second Hb-measurement during the first postoperative week was distinctly higher (median: Hb 9.75 g/dL (min/max: 7.0/12.10)) compared with untreated anemic patients in Group I (median: Hb 9 g/dL (min/max: 5.8/11.9)) (*P* < 0.05). Women with a preoperative Hb level ≥ 12 < 13 g/dL showed an Hb nadir < 9 g/dL during the first postoperative week in only 22% (5/22) compared with 44% (7/16) in Group I.

In Group II there was little correlation between the time point of ESA-/iron-substitution and preoperative Hb-course. The correlation coefficient was 0.108.

### 3.3. Reliability of the HemoCue Hemoglobinometer

**Figure 5:**
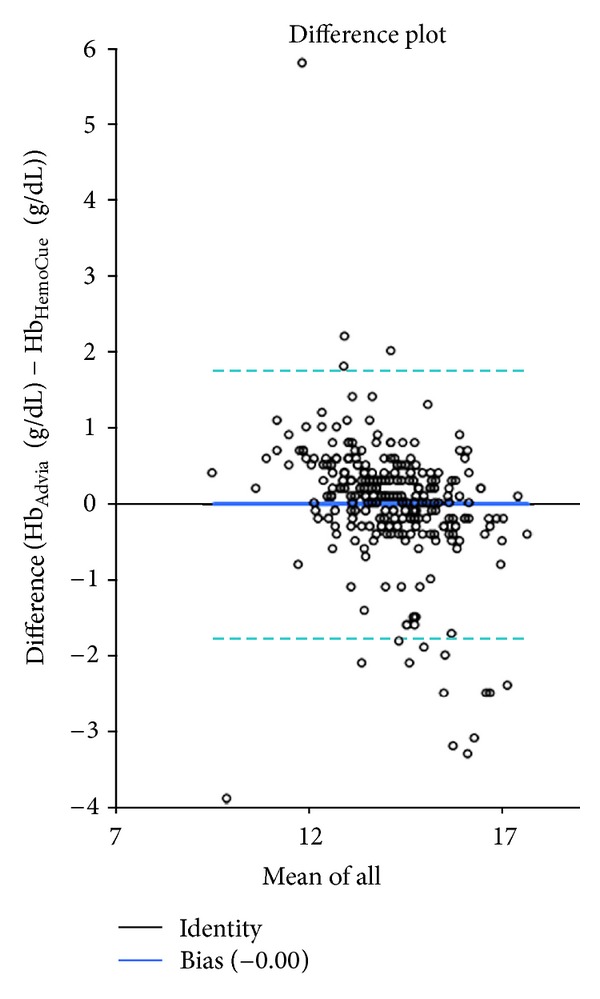
Reliability of HemoCue (NewMedX, Vienna, Austria) hemoglobinometer (*n* = 233). Bland-Altman plot of the mean bias and the limits of agreement between Hb _Advia_ and Hb _HemoCue_. The bias is represented in plain line. The limits of the agreement are illustrated by dashed lines.

In Group II the reliability of the HemoCue (NewMedX, Vienna, Austria) hemoglobinometer showed Cronbach's alpha of 0.885 and Pearson's correlation coefficient of 0.82. Bland-Altman plot is illustrated in Figure 5.

### 3.4. Differential Diagnosis of Preoperative Anemia

All in all 56 patients (Group I: *n* = 24, Group II: *n* = 32) of 335 included study participants showed preoperative anemia. 8 patients (=14.3%) had microcytic (mean MCV 77.86 fl ± 2.24 and mean MCH 26.39 pg ± 2.19) and 48 patients (=85.7%) had normocytic (mean MCV 87.61 fl ± 5.01 and mean MCH 29.48 fl ± 2.14) anemia. No case of macrocytic anemia was detected.

According to the Thomas-plot, of 56 patients with preoperative anemia, 71.4% (*n* = 40) had ACD, 19.6% (*n* = 11) had latent ID, and 9% (*n* = 5) had manifest ID. No one showed combined functional ID and ACD.

Vitamin-B12 deficiency was found in 10 patients (=3%) (mean 215.1 pg/mL ± 32.24). 9 patients (=2.8%) showed folic-acid deficiency (mean 2.53 ng/mL ± 0.27). No one presented macrocytic anemia. 4 women had combined folic-acid deficiency and latent ID, one woman combined folic-acid deficiency and manifest ID, and one man showed combined vitamin-B12 deficiency and ACD.

**Figure 6:**
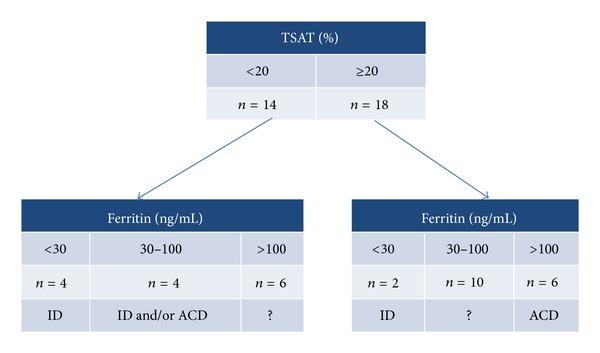
Anemic patients in Group II (*n* = 32). 6 patients with ferritin >100 ng/mL and TSAT <20%, and 10 patients with ferritin 30–100 ng/mL and TSAT ≥ 20% could not be classified with these traditional biomarkers of iron metabolism (proposed parameters in NATA guidelines). TSAT: transferrin saturation; ID: iron deficiency; ACD: anemia of chronic disease; NATA: Network for Advancement of Transfusion Alternatives.

Anemic patients in Group II (*n* = 32) were used to compare the Thomas-plot with TSAT and ferritin (recommended by NATA guidelines) as biochemical markers for the diagnosis of preoperative anemia. According to NATA guidelines, 6 patients showed ID (ferritin <30 ng/mL and/or TSAT < 20%), 6 were found to have ACD (ferritin > 100 ng/mL and/or TSAT ≥ 20%), and 4 had ID and/or ACD (ferritin 30–100 ng/mL and/or TSAT < 20%). 6 patients with ferritin >100 ng/mL and TSAT < 20% and 10 patients with ferritin 30–100 ng/mL and TSAT ≥ 20% could not be classified with these traditional parameters of iron metabolism (Figure 6). 

Using the Thomas-plot, 24 patients were found with ACD, 5 patients showed latent ID, and 3 patients had manifest ID. No one presented combined functional ID and ACD.

### 3.5. Perioperative Transfused Red Blood Cell (RBC) Units (Table 1)

 *All Patients*. In Group I (*n* = 101), 8% (8/101) of all patients received a total of 27 RBC units. Postoperatively, two patients received 1, two patients 2, and one patient 3 RBC units. All of them showed preoperative anemia. One man with preoperative anemia (Hb 11.1 g/dL) presented after knee arthroplasty a distinct hematoma on the operated lower limb and received 6 blood units postoperatively. One woman with preoperative anemia (Hb 11 g/dL) had intraoperative blood loss during knee reimplantation. Peri- and postoperatively she received 7 RBC units. One woman with preoperative Hb of 13.2 g/dL and ferritin of 10 ng/mL showed enhanced bloody ichor from wound drainage after hip arthroplasty. Postoperatively she received 5 RBC units.

In Group II (*n* = 234), 6% (15/234) of all patients received a total of 35 RBC units. Postoperatively three patients (1 with preoperative anemia) received 1, nine patients (4 with preoperative anemia) 2, and one patient with preoperative anemia 3 RBC units. One woman with a preoperative Hb of 14.1 g/dL had intraoperative blood loss during hip arthroplasty. Peri- and postoperatively she received 6 RBC units. One man with a preoperative Hb of 14 g/dL showed enhanced bloody ichor with wound hematoma after hip arthroplasty. Postoperatively he received 5 RBC units. In total perioperative RBC units in Group II were reduced by 44%.

*Anemic Patients.* Evaluating only anemic patients in Group I (*n* = 24), 29% (7/24) received 22 RBC units. In anemic patients in Group II (*n* = 32), 19% (6/32) received 12 RBC units. In total perioperative RBC units in anemic patients in Group II were reduced by 60%.

*Therapeutic Costs (Table 2)*. In our hospital the purchasing price of one RBC unit was €135 at the time of investigation. Ferric carboxymaltose cost €27.32/100 mg and erythropoietin zeta €0.69/1000 I.U. 40000 I.U. ESA was free of charge, due to an agreement between the hospital and the manufacturer, which was independent of the present study.

In Group I (*n* = 101), 27 RBC units cost in total €3645, that is, €36.08 per patient. In Group II (*n* = 234), 35 RBC units, 24 times 200 mg iron + 40000 I.U. ESA, and 8 times 1000 mg iron + 10000 I.U. ESA cost in total €8277.16, that is, €35.37 per patient. All in all the therapeutic costs at the time of investigation were cost neutral in both groups.

## 4. Discussion

In this study it was shown that laboratory-guided therapeutic intervention in patients with preoperative anemia raises their perioperative Hb level and reduces the perioperative need for blood.

The prevalence data of perioperative anemia described in the literature vary due to different definitions of anemia, different methods, and different patient collectives studied, with age, comorbidities, and nutritional behavior as cofounders [19].

Using the definition of the World Health Organization, an Hb concentration lower than 12 g/dL in women and 13 g/dL in men is diagnosed as anemia. Nevertheless these criteria might be questioned in respect of preoperative anemia management. If the transfusion risk is estimated to exceed 10%, patients with an Hb level of between 10 and 13 g/dL should be considered for iron-/ESA-substitution [5]. Present data confirm this recommendation. Women with a preoperative Hb concentration ≥ 12 < 13 g/dL also benefit from an algorithm-guided anemia diagnosis and iron-/ESA-substitution. If therapeutic interventions were performed in this group, only 22% showed a Hb nadir < 9 g/dL during the first postoperative week compared with 44% in the untreated group. 

Nevertheless, using the above-mentioned cut-offs, the results of Hb-measurements vary depending on preanalytical factors. This might be the explanation for the discrepancies in Hb levels either determined by the point-of-care testing (POCT) or the laboratory method. Our observations showed that Hb results with the HemoCue (NewMedX, Vienna, Austria) hemoglobinometer were user related and single values differed up to >2 g/dL from the reference method. Therefore POCT is not recommended to make therapeutic decisions due to insufficient accuracy [20]. Nevertheless, for practical reasons POCT is widely used. In those cases, persons using the POCT must be carefully instructed to avoid preanalytical and analytical problems.

Since anemia is just a syndrome and therapeutic interventions must be chosen depending on the underlying cause, the differential diagnosis of anemia is an important step. ID should be distinguished from other causes of anemia because it mandates specific investigation and treatment [21]. The results of this study show that the Thomas-plot is most helpful for that requirement. This diagnostic program is well adapted to differentiate classic ID from ACD and the combination of functional ID with ACD [14].

TSAT and ferritin, proposed biomarkers for preoperative anemia management in NATA guidelines, demonstrate weaknesses in distinguishing ID from other forms of anemia, especially during acute-phase reactions. Using NATA guidelines, 50% of preoperative anemia in Group II remained unexplained.

It is well known that ferritin is a positive and transferrin a negative acute-phase reactant [13–15]. Therefore acute-phase reactions influence TSAT as well as ferritin. The Thomas-plot uses sTfR as an indicator of ID, which is not increased by inflammation [6, 14, 22, 23].

For the stated reasons the Thomas-plot with sTfR/log ferritin is superior to TSAT and ferritin in the preoperative diagnosis of anemia.

For the treatment of ID we used i.v. ferric carboxymaltose. This preparation is recommended because of the low risk of adverse effects and higher tolerable maximum dose: 1000 mg iron can be infused over 15 minutes [2, 24].

The major clinical difference between oral and i.v. iron is the speed of the Hb increase (7–14 days versus 30–40 days) and the replenishment of iron stores [1, 25]. It is well known that oral iron preparations are poorly absorbed and associated with increased adverse gastrointestinal effects [26–28].

In this study the mean time point of therapeutic intervention in the substituted group was 20 days before surgery. The published data usually describe the maximum effect of i.v. iron preparations at 2 weeks [17, 19]. In this context iron-/ESA-substitution should take place at least 2 weeks before surgery.

Beside ID, vitamin-B12 deficiency and folic-acid deficiency are leading causes in anemia in the elderly [29]. Our own observations in the study collective could not confirm these data. The prevalence of vitamin-B12 deficiency was 3%, the prevalence of folic-acid deficiency 2.8%, and none of these patients had macrocytic anemia. 

In general, unexplained anemia, including preoperative macrocytic anemia, should always be considered secondary to another underlying disease [30] and subsequently clarified by a specialist in internal medicine before elective surgery. Patients with Hb level < 10 g/dL should get clinical investigations and be put under observation [31]. It is proposed that the elderly with iron-deficiency anemia should undergo gastroscopy and colonoscopy [19].

One important part of PBM is to improve diagnostic and therapeutic strategies in the preoperative setting. This reassessment of traditional diagnostic and therapeutic procedures is necessary, since the perioperative clinical outcome was shown to be hampered by blood transfusions; thus new strategies to avoid the necessity of blood products must be evaluated [4, 6–8, 10, 17, 18].

On the other hand allogenic blood units will become scarce and more expensive because of decreasing donation rates and increasing production costs [1, 3, 5, 32].

The present calculation data in our hospital show that therapeutic costs of the group with preoperative iron-/ESA-substitution are cost neutral compared with the untreated Group. Different prices in each country and the regional variation of pricing influence the calculation. The price development on allogenic RBCs must be observed accurately in the next few years.

The limitation of this study is the retrospective study design. The effect of the proposed preoperative strategy should be investigated in prospective studies. The costs of laboratory testing and staff should be included in cost accounting, if possible.

## 5. Conclusions

Algorithm-guided preoperative anemia management raises perioperative Hb level and reduces blood use.

The Thomas-plot, as a diagnostic tool, is well adapted to allow a more precise anemia classification of the preoperative setting. ID can be differentiated from ACD or combined functional ID/ACD, which becomes essential for correct therapeutic and clinical decisions.

## Figures and Tables

**Figure 4:**
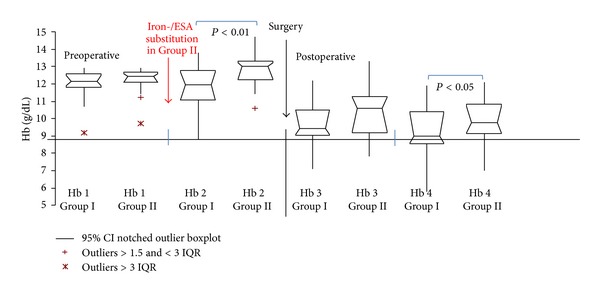
Pre- and postoperative Hb levels in anemic patients. Hb 1: first preoperative Hb-measurement in the preoperative outpatient clinic up to 3 months before surgery (Hb-median: Group I (*n* = 24): 12.15 g/dL, Group II (*n* = 32): 12.45 g/dL). Hb 2: Hb level 0–3 days before surgery (Hb-median: Group I: 11.95 g/dL, Group II: 13.0 g/dL (*P* < 0.01)). Hb 3: first postoperative Hb level (Hb-median: Group I: 9.45 g/dL, GroupII: 10.60 g/dL. Hb 4: second postoperative Hb level (Hb-median: Group I: 9.0 g/dL, Group II: 9.75 g/dL (*P* < 0.05)) 0–7 days after surgery. ESA: erythropoiesis stimulating agent, CI: confidence interval, IQR: interquartile range.

**Table 1:** Perioperative transfused allogenic RBC units.

Group I: all patients (*n* = 101)		Group II: all patients (*n* = 234)	
Men (*n* = 36)	6 RBC units	Men (*n* = 93)	15 RBC units
Women (*n* = 65)	21 RBC units	Women (*n* = 141)	20 RBC units

Total	27 RBC units	Total	35 RBC units

Group I: anemic patients (*n* = 24)		Group II: anemic patients (*n* = 32)	

Men (*n* = 1)	6 RBC units	Men (*n* = 5)	1 RBC unit
Women with Hb ≥ 12<13 g/dL (*n* = 16)	3 RBC units	Women with Hb ≥ 12 < 13 g/dL (*n* = 22)	7 RBC units
Women with Hb < 12 g/dL (*n* = 6)	13 RBC units	Women with Hb < 12 g/dL (*n* = 5)	4 RBC units

Total	22 RBC units	Total	12 RBC units

*Group I*: 8% (8/101) of all patients received 27 RBC units, respectively, 29% (7/24) of anemic patients received 22 RBC units. *Group II*: 6% (15/234) of all patients received 35 RBC units, respectively, 19% (6/32) of anemic patients received 12 RBC units. RBC: red blood cells.

**Table 2:** Therapeutic costs.

Group I (*n* = 101)			Group II ( *n* = 234)		
Product	Quantity	€/unit	Product	Quantity	€/unit
			*i.v. iron *		
			200 mg	24	27.32€ per 100 mg
			1000 mg	8	
			*ESA *		
			40000 I.E.	24	Free of charge
			10000 I.E.	8	0.69€ per 1000 I.E.
*RBC units *	27	135€	*RBC units *	35	135€

Total costs: 3645€			Total costs: 8277.16€		
Costs per patient: 36.08€			Costs per patient: 35.37€		

The data are calculated in Euro (€). Ferric carboxymaltose (Ferinject, Vifor Pharma Austria GmbH, Vienna, Austria) was used for i.v. iron substitution. Erythropoietin zeta (Retacrit, Astro-Pharma GmbH, Vienna, Austria) was used for ESA-substitution. i.v.: intravenous; ESA: erythropoiesis stimulating agent; RBC: red blood cells.
